# Disuse atrophy of articular cartilage can be restored by mechanical reloading in mice

**DOI:** 10.1007/s11033-024-09955-y

**Published:** 2024-09-27

**Authors:** Masato Nomura, Hideki Moriyama, Yoshio Wakimoto, Yasushi Miura

**Affiliations:** Department of Rehabilitation Science, Kobe University Graduate School of Health Sciences, Tomogaoka 7-10-2, Suma-ku, Kobe, Hyogo 654-0142 Japan

**Keywords:** Articular cartilage, Subchondral bone, Mechanical loading, Disuse atrophy, Reversibility

## Abstract

**Background:**

Moderate mechanical stress generated by normal joint loading and movements helps maintain the health of articular cartilage. Despite growing interest in the pathogenesis of cartilage degeneration caused by reduced mechanical stress, its reversibility by mechanical reloading is less understood. This study aimed to investigate the response of articular cartilage exposed to mechanical reloading after unloading in vivo and in vitro.

**Methods and results:**

Disuse atrophy was induced in the knee joint cartilage of adult mice through hindlimb unloading by tail suspension. For in vivo experiments, mice were subjected to reloading with or without daily exercise intervention or surgical destabilization of the knee joint. Microcomputed tomography and histomorphometric analyses were performed on the harvested knee joints. Matrix loss and thinning of articular cartilage due to unloading were fully or partially restored by reloading, and exercise intervention enhanced the restoration. Subchondral bone density decreased by unloading and increased to above-normal levels by reloading. The severity of cartilage damage caused by joint instability was not different even with prior non-weight bearing. For in vitro experiments, articular chondrocytes isolated from the healthy or unloaded joints of the mice were embedded in agarose gel. After dynamic compression loading, the expression levels of anabolic (*Sox9*, *Col2a1*, and *Acan*) and catabolic (*Mmp13* and *Adamts5*) factors of cartilage were analyzed. In chondrocytes isolated from the unloaded joints, similar to those from healthy joints, dynamic compression increased the expression of anabolic factors but suppressed the expression of catabolic factors.

**Conclusion:**

The results of this study indicate that the morphological changes in articular cartilage exposed to mechanical unloading may be restored in response to mechanical reloading by shifting extracellular matrix metabolism in chondrocytes to anabolism.

**Supplementary Information:**

The online version contains supplementary material available at 10.1007/s11033-024-09955-y.

## Introduction

Similar to muscles and bones, cartilage undergoes atrophy by disuse. Interest in the pathogenesis of cartilage thinning due to reduced mechanical stress has been growing [1]. In humans, disuse atrophy of the articular cartilage occurs 7 weeks after reduced weight bearing [2]. Therefore, disuse atrophy of joint articular cartilage may develop in patients who have been bedridden for months. However, compared with osteoarthritis (OA), which is mainly caused by the wear and tear of the articular cartilage by overuse, very few studies have examined its etiology. The first reason is its asymptomatic nature [3]. Muscle atrophy leads to impaired motor function through muscle weakness, bone atrophy increases the risk of fractures, and on the other hand, clinical problems caused by articular cartilage atrophy might be negligible. Second, disuse atrophy of the articular cartilage might recover naturally. Reloading or remobilization was reported to partially alleviate, but not completely reverse, the degeneration of articular cartilage caused by joint unloading or immobilization [4]. In addition, although reloading can restore the loss of articular cartilage matrix due to mechanical unloading, this recovery is inhibited by gentle exercise intervention, which normally has no effect [5], indicating the vulnerability of disuse-induced articular cartilage degeneration to mechanical stress. According to Brandt, some patients who are considered clinically to have posttraumatic OA may experience post-rehabilitation OA [4]. Indeed, a recent study reported that experimentally induced OA was more severe in joints with a prior unweighting period than that in healthy joints [6]. However, this study was conducted using rats, and results have not yet been confirmed in other species.

In a previous study, we reported the mechanism of articular cartilage thinning by mechanical unloading, that is, as the bone marrow space in the subchondral bone expands, cartilage resorption by osteoclasts on the bone surface extends to the cartilage; moreover, bone metabolism control factors in the deep layer of the articular cartilage lean toward bone resorption [7]. Most studies on disuse bone atrophy have focused on the cancellous bone under the growth plate or the cortical bone of the diaphysis. The response of the bone in the epiphyseal region including the subchondral bone to reloading after mechanical unloading has been limited to one report [8].

Given the above background, the primary aim of this study was to clarify whether disuse atrophy of the articular cartilage is reversible by mechanical reloading (with or without exercise intervention or surgical joint destabilization) through in vivo and in vitro experiments, and the secondary aim was to clarify morphological changes in the subchondral bone to reloading after mechanical unloading. The results of this study will help determine the appropriate rehabilitation for joint disuse.

## Materials and methods

### Experimental design and animal care

A total of 76 male C57BL/6J mice (8 weeks old, 20–26 g; Japan SLC, Shizuoka, Japan) were used in this study.

**Fig. 1:**
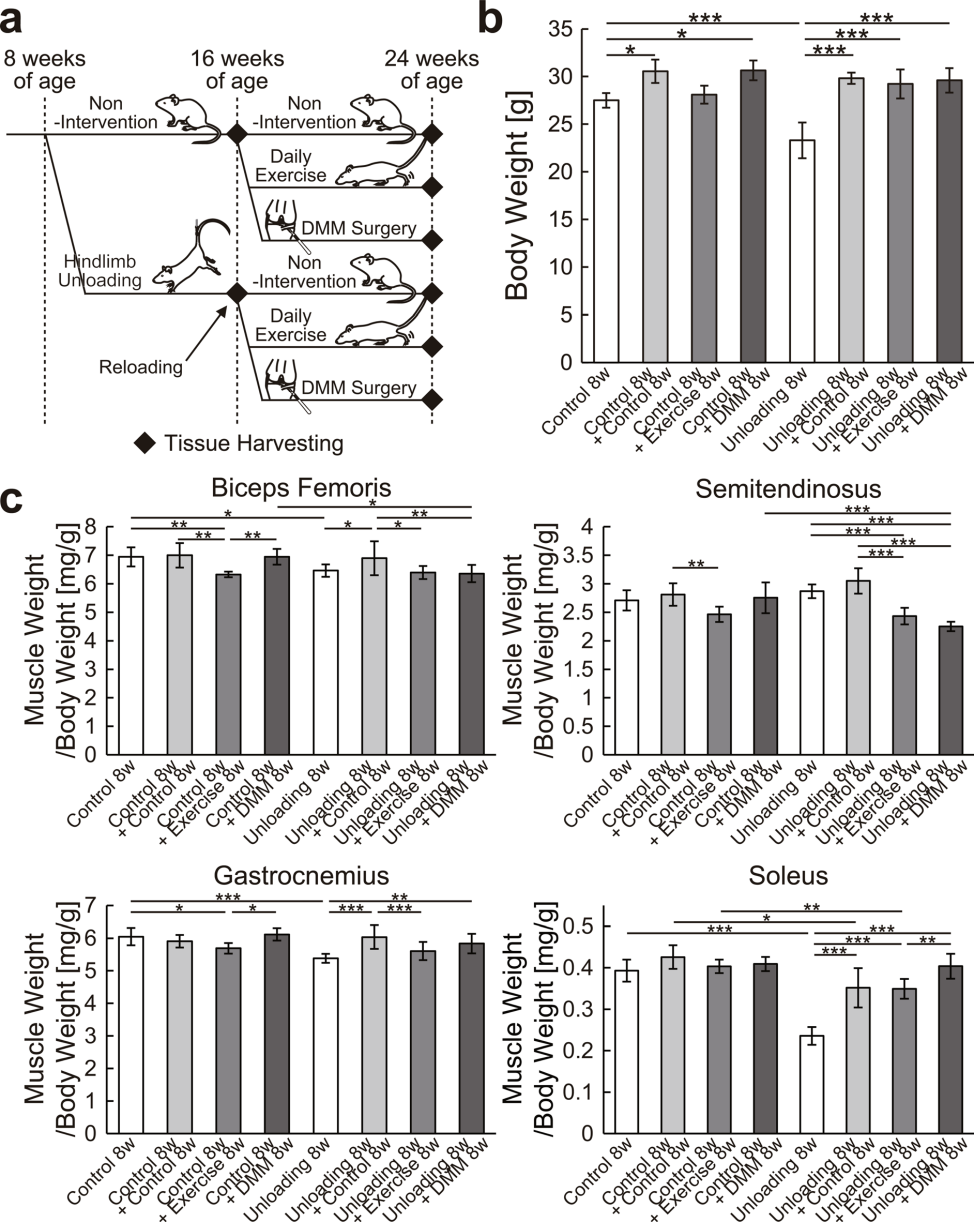
Effects of hindlimb unloading followed by reloading with or without exercise intervention or DMM surgery on the body weight and weight of muscles around the knee joint. (**a**) Experimental design of in vivo study 1. After 8 weeks of nonintervention or hindlimb unloading, mice were subjected to 8 weeks of nonintervention, daily exercise, or DMM surgery. (**b**) Body weight at the time of tissue harvesting. (**c**) Muscle weight relative to the body weight. Data are expressed as means ± SDs of four animals (**b**) or eight legs (**c**) per group. *P < 0.05, **P < 0.01, ***P < 0.001 by one-way ANOVA, followed by Tukey’s post-hoc test. DMM, destabilization of the medial meniscus

**Fig. 2:**
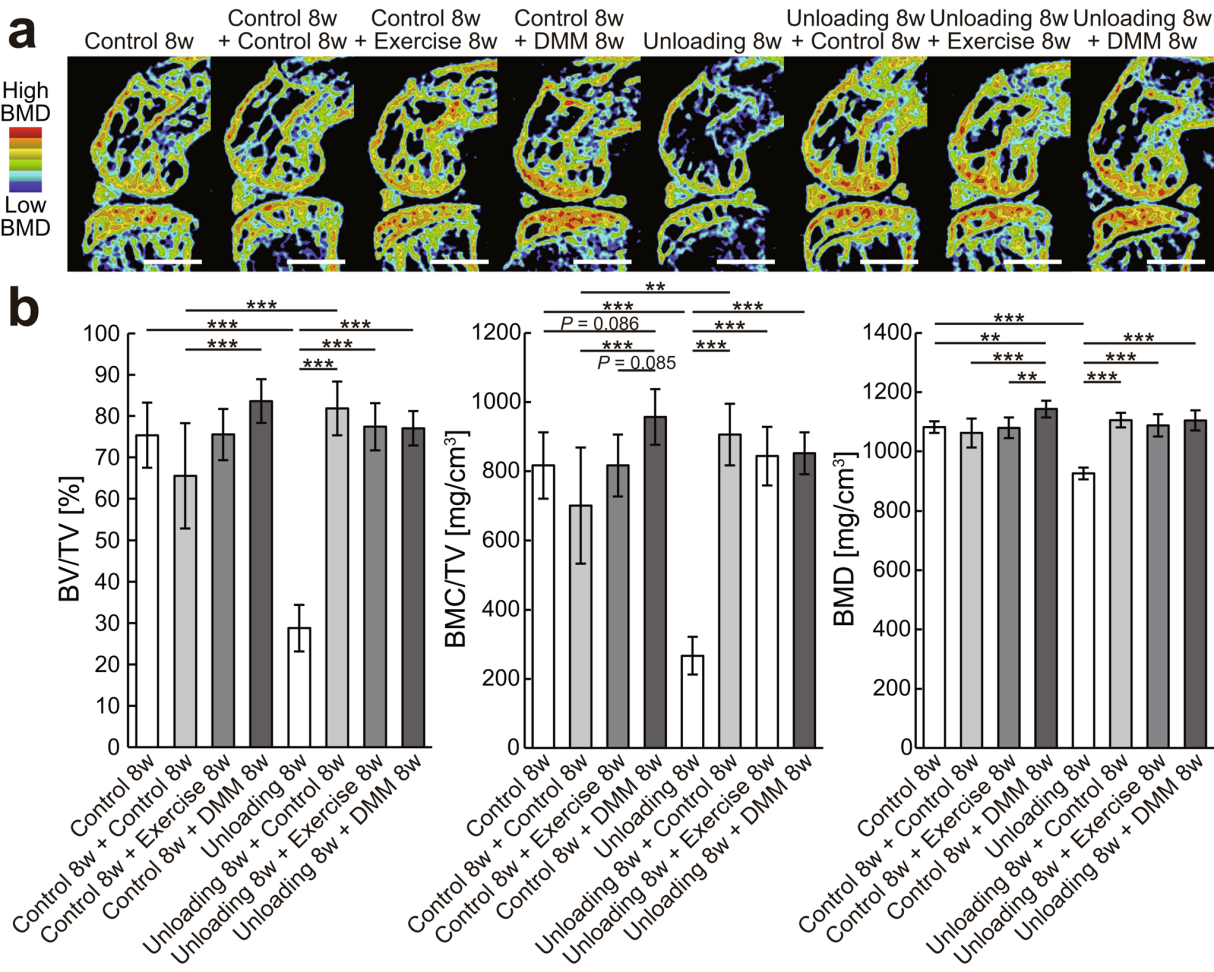
Effects of hindlimb unloading followed by reloading with or without exercise intervention or DMM surgery on subchondral bone parameters at the medial tibial plateau. (**a**) BMD images at the medial midcondylar level of the sagittal plane. Red and blue represent high and low BMD, respectively. Scale bars = 1 mm. (**b**) Quantification of subchondral bone parameters at the medial tibial plateau. Data are expressed as means ± SDs of eight joints per group. *P < 0.05, **P < 0.01, ***P < 0.001 by the one-way ANOVA, followed by Tukey’s post-hoc test. BMD, bone mineral density; DMM, destabilization of the medial meniscus

**Fig. 3:**
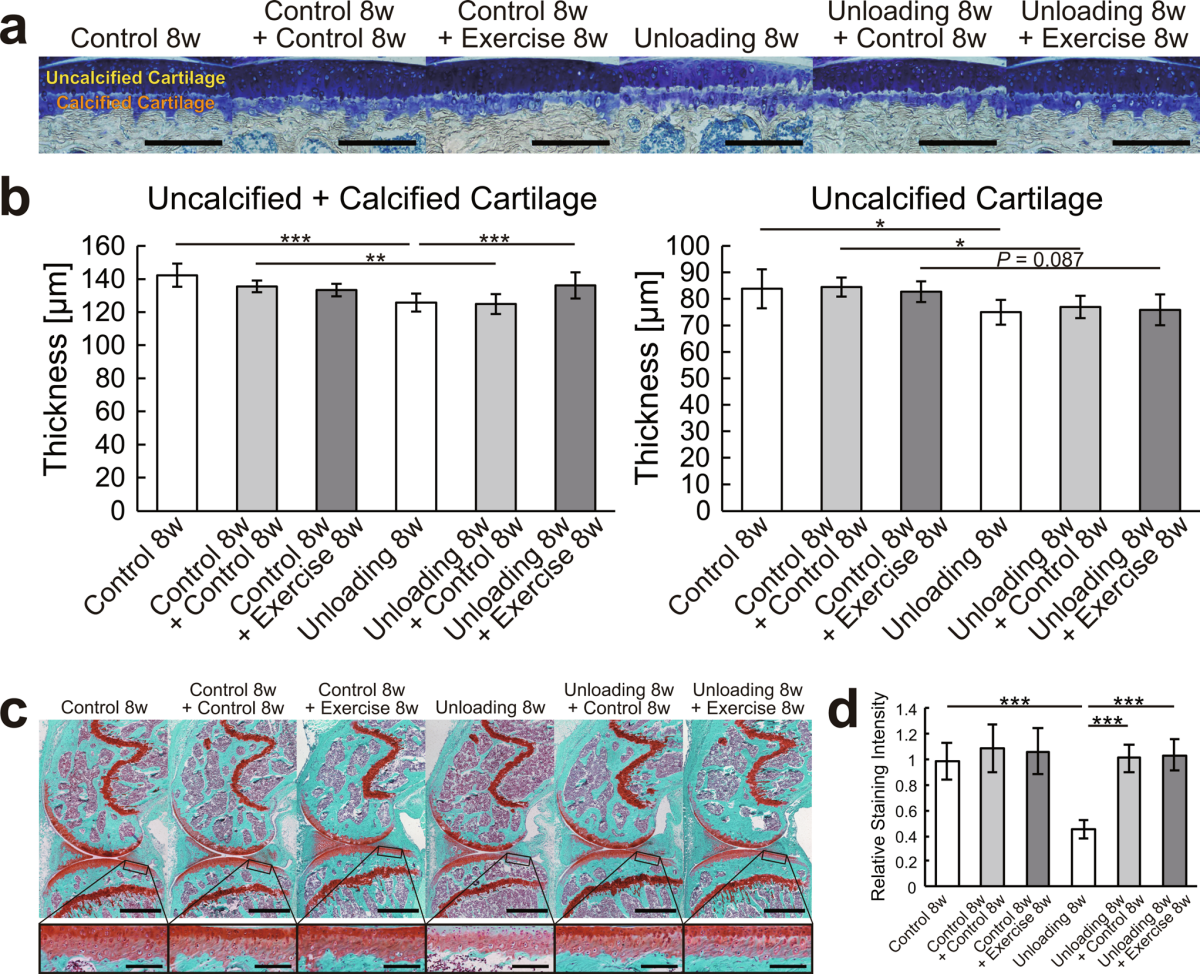
Effects of hindlimb unloading followed by reloading with or without exercise intervention on cartilage thickness and Safranin O stainability. (**a**) Representative histological images of the medial tibial plateau stained with toluidine blue. Scale bars = 200 μm. (**b**) Thickness of the uncalcified and calcified layers of the articular cartilage were measured separately at the center of the tibial plateau. (**c**) Representative histological images of the whole knee joint (upper panels) and posterior region of the tibia (lower panels; higher-magnification views of the boxed areas in the upper panels) stained with Safranin O. Scale bars = 1 mm (upper panels) and 100 μm (lower panels). (**d**) Quantification of the staining intensity for Safranin O in the uncalcified cartilage at the posterior regions of the tibia. (**b**, **d**) The average of the measurements on three slides for each joint was calculated. Data are expressed as means ± SDs of eight joints per group. *P < 0.05, **P < 0.01, ***P < 0.001 by the one-way ANOVA, followed by Tukey’s post-hoc test

**Fig. 4:**
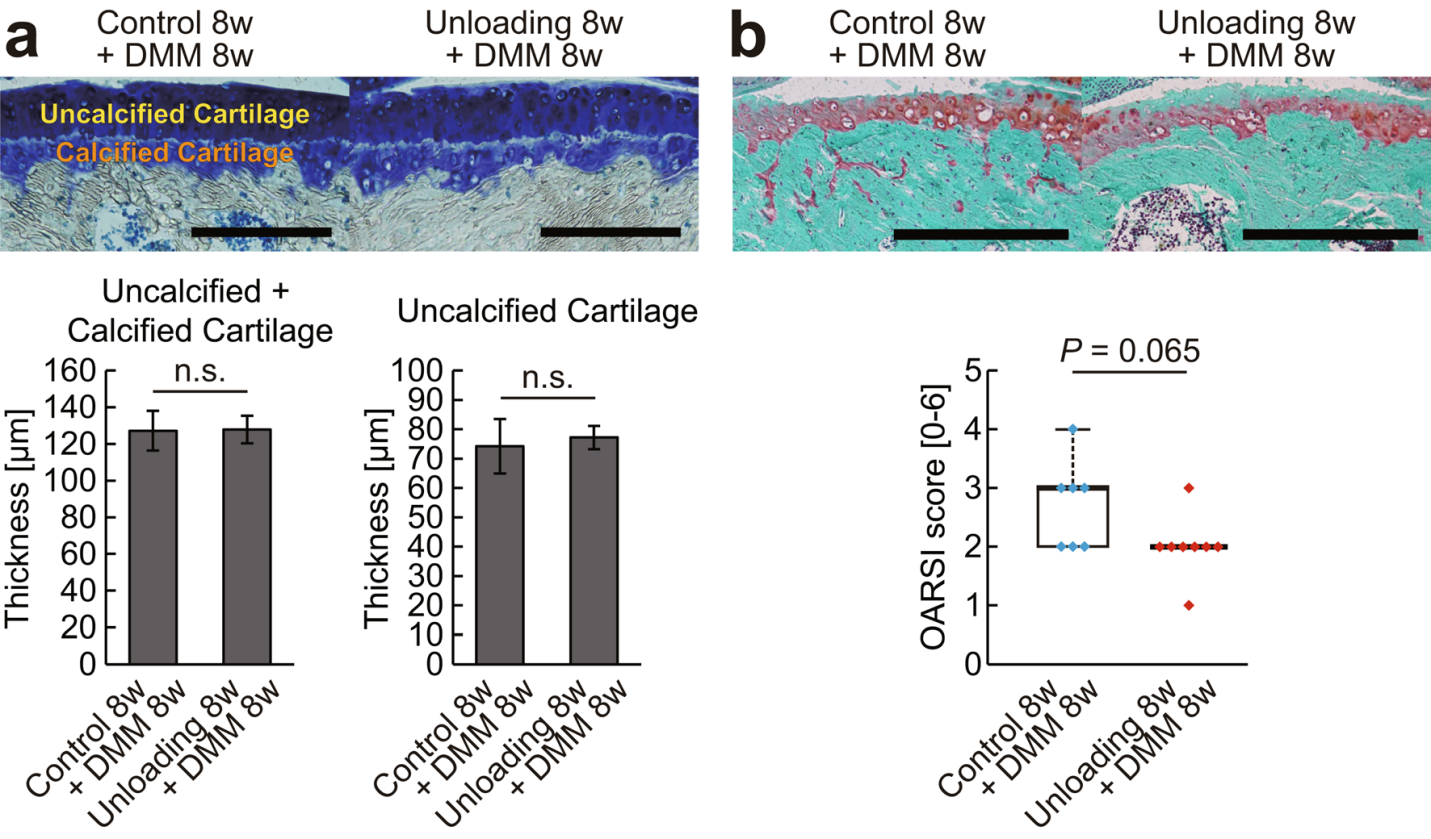
Effect of hindlimb unloading followed by reloading on OA induced by DMM surgery. (**a**) Representative histological images of the medial tibial plateau stained with toluidine blue and histomorphometric analysis of articular cartilage thickness. Scale bars = 200 μm. The average of the measurements on three slides for each joint was calculated, and data are expressed as means ± SDs of eight joints per group. n.s. indicates no significant difference by Student’s t-test. (**b**) Representative histological images of the medial tibial plateau stained with Safranin O and histopathological assessment of cartilage destruction using the OARSI scoring system. Scale bars = 500 μm. Four slides were evaluated for each joint, and the highest score was recorded. Data are expressed as medians and quartiles of eight joints per group. *P* = 0.065 by Mann–Whitney U test. DMM, destabilization of the medial meniscus; OA, osteoarthritis

The experimental design of in vivo study 1 is shown in Fig. 1a. In total, 32 mice were randomly assigned to the following eight groups of four mice each: (i) 8 weeks of normal housing, (ii) 16 weeks of normal housing, (iii) 8 weeks of normal housing followed by 8 weeks of normal housing with daily exercise intervention, (iv) 8 weeks of normal housing followed by surgical destabilization of the medial meniscus (DMM) and 8 weeks of normal housing, (v) 8 weeks of hindlimb unloading, (vi) 8 weeks of hindlimb unloading followed by reloading and 8 weeks of normal housing, (vii) 8 weeks of hindlimb unloading followed by reloading and 8 weeks of normal housing with daily exercise intervention, and (viii) 8 weeks of hindlimb unloading followed by reloading with DMM surgery and 8 weeks of normal housing. The right and left knees were used as different samples for the measurements of body weight and muscle weight (Fig. 1b and c), µCT analysis of the knee joints (Fig. 2, Supplementary Figs. 2 and 3), and histological analysis of the articular cartilage (Figs. 3 and 4).

The experimental design of in vivo study 2 is shown in Supplementary Fig. 1a. Here, 12 mice were randomly assigned to the following six groups of two mice each: (i) 6 weeks of normal housing, (ii) 2 weeks of normal housing followed by 4 weeks of normal housing with daily exercise intervention, (iii) 2 weeks of normal housing followed by DMM surgery and 4 weeks of normal housing, (iv) 2 weeks of hindlimb unloading followed by reloading and 4 weeks of normal housing, (v) 2 weeks of hindlimb unloading followed by reloading and 4 weeks of normal housing with daily exercise intervention, and (vi) 2 weeks of hindlimb unloading followed by reloading with DMM surgery and 4 weeks of normal housing. The right and left knees were used as different samples for the histomorphometric analysis of the subchondral bone (Supplementary Fig. 1b and c).

**Fig. 5:**
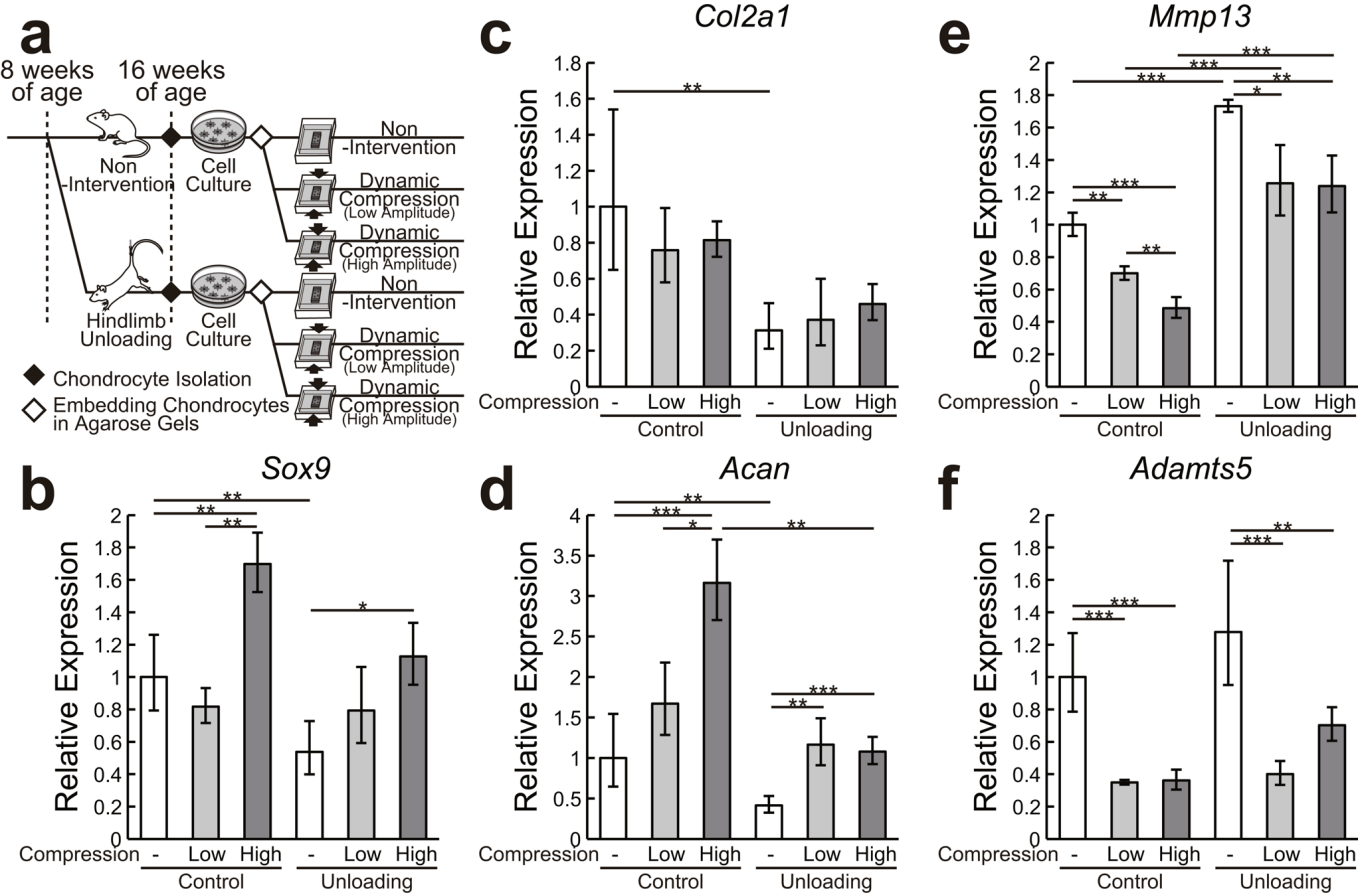
Effect of joint unloading in vivo on the responses of articular chondrocytes to mechanical stress in vitro. (**a**) Experimental design of the in vitro study. After 8 weeks of nonintervention or hindlimb unloading, chondrocytes were isolated from the knee joint cartilage. The cells were embedded in agarose gels at the second passage and then subjected to dynamic compression with strain amplitude of 8% (low) or 15% (high) at 1 Hz for 24 h. (**b**–**f**) mRNA expression levels of anabolic (*Sox9* (**b**), *Col2a1* (**c**), and *Acan* (**d**)) and catabolic (*Mmp13* (**e**) and *Adamts5* (**f**)) factors quantified by real-time polymerase chain reaction. Data are expressed as means ± SD of four wells per group. *P < 0.05, **P < 0.01, ***P < 0.001 by the one-way ANOVA, followed by Tukey’s post-hoc test

The experimental design of the in vitro study is shown in Fig. 5a. In this part, 32 mice were randomly assigned to the following two groups of 16 mice each: (a) 8 weeks of normal housing and (b) 8 weeks of hindlimb unloading. Articular chondrocytes were isolated from the hindlimbs and used for real-time polymerase chain reaction (PCR) analysis of the changes in the expression level of anabolic and catabolic factors in response to dynamic compression (Fig. 5b and c).

The mice were kept in a temperature-controlled environment (22 °C) with humidity of 55% ± 5% and 12-h light/dark cycle. Water and food were available ad libitum, and activity was not restricted.

### Hindlimb unloading by tail suspension

Hindlimb unloading was performed by tail suspension following a modified method based on the traditional NASA Morey–Holton design [9]. Briefly, a sterile steel wire was inserted into an intervertebral space of the tail and shaped into a ring for later suspension. By connecting the tail ring by a string to a track hanging from the ceiling, the animals could roam freely around the cage. Throughout the experimental period, the head-down tilt angle was monitored every day and remained at approximately 30°, so that 50% of the body weight was distributed to the forelimbs [10].

### Exercise intervention

A treadmill device (MK-680, Muromachi Kikai Co., Ltd., Tokyo) was used for the exercise intervention. According to a previous study [11], mice were forced to run at a speed of 17 m/min with a gradient of 20% for 40 min once a day, seven days a week (every day). The first 4 days were used as a generalization period, during which the speed and time were gradually increased to the above values. Running at 17 m/min is considered an intense exercise that requires > 90% of the maximum oxygen intake for mice [12].

### OA induction by DMM surgery

OA was surgically induced in mice by DMM surgery of both knee joints as described previously [13, 14]. Briefly, under anesthesia with isoflurane (1.5–3.0%, 200–300 ml/min), the hair was shaved, the skin and joint capsule were incised, and the medial meniscotibial ligament was resected. After the joint capsule and skin were sutured, 0.05 mg/kg buprenorphine was administered subcutaneously every 8–12 h during and up to 48 h after surgery to relieve postoperative pain.

### Tissue harvesting

At the end of the experimental period of in vivo studies 1 and 2, the animals in each group were euthanized by exsanguination under general anesthesia (1.5–3.0% isoflurane at 200–300 mL/min), and pain was controlled by subcutaneous injection of 0.05 mg/kg buprenorphine. Knee joints were removed by dissection.

### µCT analyses

µCT analyses were performed as detailed previously [7]. Briefly, the whole knee joint samples were scanned by a micro 3D X-ray CT system (R_mCT2; Rigaku, Tokyo, Japan) at an isotropic voxel resolution of 10 μm (X-ray tube potential, 90 kV; current, 160 µA; scan time, 3 min per sample). 3D images were reconstructed and analyzed with TRI/3D-BON software (Ratoc, Tokyo, Japan). Bone volume density (bone volume [BV]/total volume [TV]), bone mineral content (bone mineral content [BMC]/TV), and bone mineral density (BMD; BMC/BV) were measured on the epiphyseal regions of the proximal tibia. The µCT scanned samples were then also used for histological analyses.

### Histological preparation

Undecalcified frozen sections were prepared according to the method described by Kawamoto [15]. The whole knee joint samples were freeze-embedded with 5% carboxymethyl cellulose gel. Blocks were cut into slices from the medial side of the knees, and 5-µm sagittal sections were prepared at the level, which is 50–200 μm lateral from the level that the medial meniscus separates into the anterior and posterior horns.

### Measurement of cartilage thickness

According to the modification of our previous method [7], articular cartilage thickness was measured on the digitized images of histological sections stained with toluidine blue, which provides excellent color discrimination between the bone and calcified cartilage and distinct basophilic line that marks the location of the tidemark [16]. Briefly, at the center of the tibial plateau, which has a high proportion of uncalcified cartilage [7] similar to other species, a 500 μm-long stretch of the cartilage surface was defined, and the uncalcified and calcified cartilage areas under this stretch were measured separately. The thickness of each layer was calculated by dividing the area by 500 μm. Total cartilage thickness was the sum of the thickness of uncalcified and calcified layers. The mean thickness for each specimen was derived by averaging measurements from four slides.

### Quantification of cartilage matrix stainability

In our previous study, the loss of the articular cartilage matrix of the tail-suspended mouse was observed in the posterior region of the knee joint [7]. Therefore, cartilage matrix content was quantified at the posterior region of the tibial plateau. The sections were fixed with 100% ethanol for 2 min followed by 4% PFA/PBS for 30 min and decalcified with 10% EDTA/PBS for 60 min. The digitized images were converted to grayscale images with Adobe Photoshop CS2 (Adobe Systems, San Jose, CA, USA). The mean of the pixel gray values (range, 0–255) was measured with Image J 1.50b (National Institutes of Health, Bethesda, MD, USA).

### Osteoarthritis research society international (OARSI) scoring

The OARSI scoring system [17], on a scale from 0 (normal) to 6 (severe), was used for the semiquantitative evaluation of the cartilage lesion at the medial tibial plateau after DMM surgery. The highest score among four slides was recorded.

### Chondrocyte isolation and culture

At the end of the experimental period of the in vitro study, the mice were euthanized by exsanguination under anesthesia, and primary chondrocytes were isolated from the femoral head, femoral condyles, and tibial plateau as detailed previously [18]. The cartilage samples (excluding the subchondral bone that appears brown) were rinsed with phosphate-buffered saline, and chondrocytes were isolated from the cartilage using 0.4% collagenase (034-22363; FUJIFILM Wako Pure Chemical Co., Osaka, Japan) overnight at 37 °C. These chondrocytes were seeded in a 35-mm cell culture dish (353801; BD Falcon, Tokyo, Japan) and cultured in Dulbecco’s Modified Eagle Medium–Ham’s F12 medium (042-30555; FUJIFILM Wako Pure Chemical Co.) supplemented with 10% fetal bovine serum (Gibco 12,483,020; Thermo-Fisher Scientific, Inc., MA, USA), 50 units/mL penicillin, and 50 µg/mL streptomycin (168-23191; FUJIFILM Wako Pure Chemical Co.) in an incubator maintained at 37 °C with 5% CO_2_. The medium was changed every 3 days. At up to 80–90% confluency, the chondrocytes were harvested with a trypsin–ethylenediaminetetraacetic acid solution (209-16941; FUJIFILM Wako Pure Chemical Co.) for passage.

### Preparation of chondrocyte–agarose constructs and dynamic compression intervention

Chondrocyte–agarose constructs were prepared as described previously [19]. Briefly, equal volumes of 4% (w/v) agarose/PBS and 4 × 10^6^ cells/mL of the second passage chondrocyte suspension were mixed and poured into a chamber mold (TB-CH-3.5GS; Strex Inc., Osaka, Japan) and gelled. A conditioned medium was added on top of the gel and replaced once a day. After 1 week, dynamic compression with a strain amplitude of 8% (low amplitude) or 15% (high amplitude) at 1 Hz for 24 h was applied to the chondrocyte–agarose constructs using the Strex device (STB-140; Strex Inc., Osaka, Japan) (Supplementary Videos 1 and 2).

### RNA isolation and real-time PCR

Immediately after dynamic compression, total RNA was isolated from the chondrocyte–agarose constructs using Qiagen RNeasy Plus Universal Mini kit (#73404, QIAGEN, Venlo, Holland), according to the manufacturer’s instructions. The purity and concentration of the extracted total RNA were measured using a BioPhotometer D30 (Eppendorf, Hamburg, Germany).

Reverse-transcription and real-time PCR were performed using the StepOne Real-Time PCR system (Thermo-Fisher Scientific Inc.) with the TaqMan™ Fast Virus 1-Step Master Mix (Thermo-Fisher Scientific Inc.) and Gene Expression Assays (Applied Biosystems, CA, USA) for sex-determining region Y-box 9 (SOX9) mRNA (*Sox9*; Mm00448840_m1), collagen type II alpha1 mRNA (*Col2a1*; Mm01309565_m1), aggrecan mRNA (*Acan*; Mm00545794_m1), matrix metallopeptidase 13 (MMP13) mRNA (*Mmp13*; Mm00439491_m1), disintegrin-like and metallopeptidase with thrombospondin type 1 motif 5 (ADAMTS5) mRNA (*Adamts5*; Mm00478620_m1), and 18 S ribosomal (18s) mRNA (*18s*: Mm03928990_g1). Their expression levels were analyzed using the 2^−Δ Δ CT^ method [20, 21] and normalized to *18s* levels [22].

### Statistical analysis

Statistical analyses were performed with EZR (Saitama Medical Center, Jichi Medical University, Saitama, Japan), which is a graphical user interface for R (The R Foundation for Statistical Computing, Vienna, Austria) [23]. First, all data were checked for normality using the Shapiro–Wilk test. When normality was observed in all assays, the results were compared between two groups using Student’s t-test or compared among three or more groups with the analysis of variance test followed by the Tukey HSD test. These values are presented as means ± standard deviations (SDs). The OARSI score was compared with the Mann–Whitney U test. The values are presented as medians and quartiles. *P*-values < 0.05 were considered significant.

## Results

### Body and muscle weight

The results of body weight measurements are shown in Fig. 1b. Although the body weight of mice significantly decreased after hindlimb unloading (*P* < 0.001), it recovered to the same level as the control after subsequent reloading. Similar changes were observed in the exercise intervention and DMM surgery groups.

The results of measuring the wet weight of the muscles around the knee joint are shown in Fig. 1c. The wet weights of the biceps femoris, gastrocnemius, and soleus muscles were significantly decreased by hindlimb unloading (*P* < 0.05) and largely recovered by subsequent reloading; however, the recovery was partially hindered by exercise intervention or DMM surgery. The wet weight of the semitendinosus muscle did not change significantly by hindlimb unloading and subsequent reloading but was significantly decreased by the combination of reloading and exercise intervention or DMM surgery (*P* < 0.001).

### Cartilage thickness

The histological observation and results of thickness measurements of the articular cartilage at the center of the tibial plateau are shown in Figs. 3a and b, and 4a.

Hindlimb unloading significantly decreased the combined thickness of the uncalcified and calcified layers of the articular cartilage (*P* < 0.001), and subsequent reloading recovered it to the same level as the control (*P* < 0.01). However, the group with exercise intervention showed significantly higher values ​​than the group without it (*P* < 0.001) and showed recovery to the same level as mice that received continued normal housing and the same intervention.

The thickness of uncalcified layer was also significantly decreased by hindlimb unloading (*P* < 0.05) and was not recovered by subsequent reloading (*P* < 0.05). However, the group that received exercise intervention showed no significant difference compared with the group that received continued normal housing and the same intervention (*P* = 0.087).

### Cartilage matrix stainability

The histological observation at the posterior region of the tibial plateau and the results of quantification of the Safranin O staining intensity are shown in Fig. 3c and d. The staining intensity was significantly decreased by hindlimb unloading (*P* < 0.001) and significantly increased by reloading (*P* < 0.001) regardless of the exercise intervention, restoring to the same level as control.

### OA severity

The histological observation and severity of OA induced by DMM surgery are illustrated in Fig. 4b. No significant difference in the OARSI score was found between the groups that underwent DMM surgery after normal housing and after hindlimb unloading followed by reloading (*P* = 0.065). As shown in Fig. 4a, no significant differences in the full thickness of articular cartilage and thickness of the uncalcified layer were found between the groups.

As supplemental information, no subluxation/dislocation of the patella was found, which is an abnormal finding after DMM surgery [24]. However, the medial deviation of the MM was observed, which is a normal finding of the model (Supplementary Fig. 2).

### Subchondral bone density

BMD images and bone morphometry results at the medial condylar level of the knee joint are shown in Fig. 2. BV/TV, BMC/TV, and BMD (BMC/BV) were significantly decreased by hindlimb unloading (*P* < 0.001) and significantly increased by subsequent reloading with or without exercise intervention or DMM surgery (*P* < 0.001). Furthermore, BV/TV and BMC/TV in joints subjected to unloading followed by reloading were significantly higher than those continuously grounded (*P* < 0.001 and 0.01, respectively). This abnormal finding of the subchondral bone observed after unloading followed by reloading (Fig. 2) was also observed when the intervention period was shortened (Supplementary Fig. 1).

BMD images and results of bone morphometry at the lateral condylar level of the knee joint are shown in Supplementary Fig. 3. The results were nearly the same as those at the medial condylar level; however, no bone increase was observed in the group that underwent DMM surgery after normal housing, indicating successful localization of the pathology to the medial condyle.

### mRNA in vitro expression

The results of measuring the mRNA expression levels of anabolic (*Sox9*, *Col2a1*, *Acan*) and catabolic factors (*Mmp13*, *Adamts5*) in chondrocytes exposed to mechanical stimulation in vitro are shown in Fig. 5. Compared with normal chondrocytes, articular chondrocytes isolated from joint tissue exposed to mechanical unloading showed significantly decreased expression levels of *Sox9*, *Col2a1*, and *Acan* (*P* < 0.01) and significantly increased expression level of *Mmp13* (*P* < 0.01). Dynamic compression with high amplitudes significantly increased the expression levels of *Sox9* and *Acan* in both normal and unloaded chondrocytes (*P* < 0.05), whereas the expression level of *Acan* was significantly lower in unloaded chondrocytes than in normal chondrocytes (*P* < 0.01). In addition, dynamic compression with high and low amplitudes significantly decreased the expression levels of *Mmp13* and *Adamts5* in both normal and unloaded chondrocytes (*P* < 0.05), whereas the expression level of *Mmp13* was significantly higher in unloaded chondrocytes than in normal chondrocytes (*P* < 0.001).

## Discussion

In this study, the response of articular cartilage and subchondral bone to reloading after mechanical unloading was investigated. Unloading-induced matrix loss and articular cartilage thinning were fully or partially restored by reloading, and exercise intervention enhanced this recovery (Fig. 3). Furthermore, in chondrocytes isolated from joints exposed to unloading, in vitro dynamic compression increased the expressions of cartilage anabolic factors but suppressed those of cartilage catabolic factors, similar to healthy chondrocytes (Fig. 5). These data indicate that articular chondrocytes exposed to mechanical unloading maintain the potential to restore morphological changes by shifting extracellular matrix metabolism toward an anabolic state in response to reloading. The severity of OA caused by joint instability was not affected by prior unweighting (Fig. 4); however, reloading after unloading induced the formation of large amounts of subchondral bone (Fig. 2), which might be an early symptom of OA.

No consensus has been established regarding the reversibility of disuse articular cartilage atrophy. A human study reported that proteoglycan contents in the ipsilateral knee cartilage of patients with unilateral ankle fractures were altered by 6 weeks of bilateral crutch walking, and this change persisted up to 1 year after reweighting [25]. In addition, animal studies have shown that articular cartilage degeneration caused by unloading or joint immobilization can be at least partially reversed by reloading or remobilization. However, many studies have reported that the degeneration was not completely reversed [4]. In the present study, the loss of the articular cartilage matrix due to unloading was completely restored by reloading, whereas thinning was not completely restored (Fig. 3). Discrepancies among studies might be due to variations in the duration of pathogenesis and recovery. Although the reported half-life of proteoglycan turnover was 8 days [26], the half-life of aggrecan fragments in tissues ranged from 3 to 25 years [27]. Therefore, a sufficient recovery period is required to completely restore the altered morphology, depending on the duration of the pathogenesis. Because exercise intervention promoted recovery (Fig. 3), active rehabilitation might be effective. However, the amount of load should be carefully decided, considering that another study showed that exercise inhibited it [5].

Chondrocytes, the only cellular component present in the articular cartilage, control the extracellular matrix metabolism primarily through the synthesis and degradation of collagen fibers and proteoglycans [28]. Applying periodic mechanical stress to chondrocytes in vitro, which mimics stimuli experienced in vivo, was found to enhance anabolic effects but suppress catabolic effects [29]. To the best of our knowledge, this study was the first to show that articular chondrocytes maintain such a mechanotransduction function even after disuse (Fig. 5). These data strongly support the notion that the articular cartilage may recover through mechanical reloading, even after disuse alteration. However, the magnitude of their response to mechanical stress was, in some cases, less than those of healthy chondrocytes, raising suspicions of impaired function and calling for further research.

A close relationship was reported between articular cartilage and subchondral bone [30]. Previously, we demonstrated that unloading-induced thinning of the articular cartilage may be related to subchondral bone atrophy [7]. A subsequent study reported that this atrophy of the subchondral bone can be restored by physiological loading [8]. Similar to this study, the loss of subchondral bone density due to mechanical unloading was reversed by reloading in the present study, with the difference that greater bone mass than normal was formed, during two experimental periods (Fig. 2, Supplementary Fig. 1). Because subchondral bone sclerosis is an early OA symptom [24], longer experimental period may lead to the spontaneous onset of OA.

OA is mainly caused by excessive mechanical stress on the joints [31]. In addition, a previous study using rats reported that OA caused by joint instability was more severe in joints with prior unloading exposure than that in normal joints [6]. In the present study, the severity of OA caused by joint instability was not affected by prior unweighting (Fig. 4). This discrepancy with the results of a previous study may be due to differences in animal species and/or experimental duration.

The main limitation of this study is that we were unable to evaluate the amount of load applied to the articular cartilage in the observed area. Thus, whether weight bearing was applied to the hind limbs in mice that were reloaded after hindlimb unloading, as in healthy mice, is unclear. Although the body weight returned to normal, the weight of some muscles around the knee joint remained decreased and did not recover (Fig. 1). If this indicates that the hindlimbs were not able to bear weight, it may have influenced the degree of OA caused by DMM surgery and led to a difference from the results of a previous study [6]. The limitations include a small sample size and limited statistical power, which prevents us from ruling out chance findings. It is open for discussion whether the results represent a random finding due to the small sample size or are, for example, due to the remaining muscle atrophy after reloading.

## Conclusion

This study provided data supporting the reversibility of disuse-induced cartilage degeneration based on in vivo and in vitro experiments. The data support the importance of active joint rehabilitation in patients on a chronic bedridden state. However, excessive formation of the subchondral bone observed in this study might be an early symptom of OA. Therefore, careful consideration is required when deciding the amount of reloading, and further research on treatment strategies is warranted.

## Electronic supplementary material

Below is the link to the electronic supplementary material.

Supplementary Fig. 1: Effects of hindlimb unloading followed by reloading with or without exercise intervention or DMM surgery on subchondral bone histomorphology at the medial tibial plateau. A supplementary short-term experiment. (a) Experimental design of in vivo study 2. After 2 weeks of nonintervention or hindlimb unloading, mice were subjected to 4 weeks of nonintervention, daily exercise, or DMM surgery. (b) Representative histological images of the medial tibial plateau stained with Safranin O. Scale bars = 1 mm. (c) Quantification of subchondral bone density at the medial tibial plateau. The bone and total area were measured separately in the epiphyseal region on the images, and the percentage of the bone area relative to the total area was calculated. Data are expressed as means ± SD of 4 joints per group. **P < 0.01, ***P < 0.001 by the one-way ANOVA, followed by Tukey’s post-hoc test. DMM, destabilization of the medial meniscus.

Supplementary Fig. 2: Three-dimensional images of the whole knee joints obtained by microcomputed tomography. Frontal view. Scale bars = 1 mm. For all samples, no patella displacement was observed.

Supplementary Fig. 3: Effects of hindlimb unloading followed by reloading with or without exercise intervention or DMM surgery on subchondral bone parameters at the lateral tibial plateau. (a) BMD images at the lateral midcondylar level of the sagittal plane. Red and blue represent high and low BMDs, respectively. Scale bars = 1 mm. (b) Quantification of subchondral bone parameters at the lateral tibial plateau. Data are expressed as means ± SD of eight joints per group. *P < 0.05, **P < 0.01, ***P < 0.001 by the one-way ANOVA, followed by Tukey’s post-hoc test. BMD, bone mineral density.

Supplementary Video 1: Dynamic compression of chondrocyte–agarose constructs with strain amplitude of 8% at 1 Hz.

Supplementary Video 2: Dynamic compression of chondrocyte–agarose constructs with strain amplitude of 15% at 1 Hz.

## Data Availability

No datasets were generated or analysed during the current study.
