# Correction: Disuse atrophy of articular cartilage can be restored by mechanical reloading in mice

**DOI:** 10.1007/s11033-024-10081-y

**Published:** 2024-11-16

**Authors:** Masato Nomura, Hideki Moriyama, Yoshio Wakimoto, Yasushi Miura

**Affiliations:** Department of Rehabilitation Science, Kobe University Graduate School of Health Sciences, Tomogaoka 7-10-2, Suma-ku, Kobe, Hyogo 654-0142 Japan

**Correction to: Molecular Biology Reports (2024) 51:1018**

10.1007/s11033-024-09955-y

The original article has been published with incorrect citation and order of figures from figs. 2, 3 and 4.

And the references 3, 14, 25, 26, and 28 have been updated with correct citation information in reference list. Below are the correct information of these references.

The original article has been corrected.
